# Supplementary material for: Acute RyR1 Ca2+ leak enhances NADH-linked mitochondrial respiratory capacity
Source: Nat Commun. 2021 Dec 10;12:7219. doi: 10.1038/s41467-021-27422-1 (PMC8664928; doi:10.1038/s41467-021-27422-1)
Supplement: Supplementary file 1 — Supplementary information [file 41467_2021_27422_MOESM1_ESM.pdf]

Supplementary information for

[Acute RyR1 Ca^{2+} leak enhances NADH-linked mitochondrial respiratory capacity]

Supplementary Figures

Supplementary Fig. 1

a

	MICT	SIT
Age (yrs)	23 (± 2)	25 (± 4)
Body mass (kg)	73 (± 5)	78 (± 7)
Height (cm)	181 (± 8)	178 (± 5)
VO_2 peak (ml/min/kg)	54 (± 6)	53 (± 6)

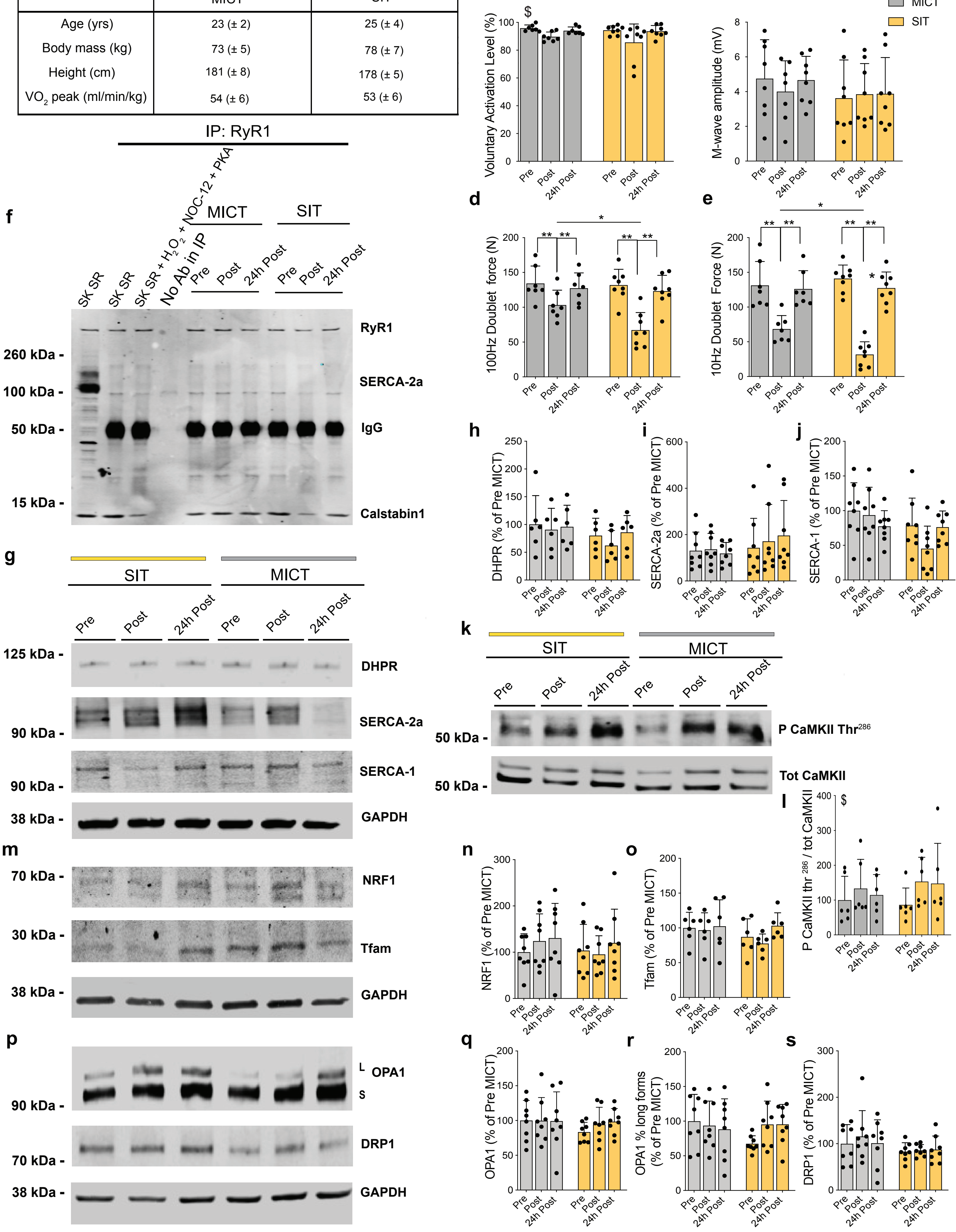

Supplementary Fig. 1. Participant characteristics, knee extensor neuromuscular function, and sarcoplasmic reticulum and mitochondrial proteins in human muscle

a Physiological characteristic of the volunteers involved in the study; n = 8 participants per group. Values are mean ± SD.
b-e Knee extensor maximal voluntary activation level (**b**), M-wave amplitude (**c**) and evoked force in response to paired stimuli at 100 Hz and 10Hz applied to the femoral nerve (d and e, respectively); n = 8 participants per group. 2-way ANOVA followed by Sidak's multiple comparisons test (d and e).
f Immunoblots of non-immunoprecipitated and immunoprecipitated RyR1 samples, and immunoblots of RyR1, SERCA-2a and calstabin1. The IgG are shown to distinguish the immunoprecipitated samples from the positive control for SERCA-2a (first lane). SK SR: skeletal muscle sarcoplasmic reticulum vesicle. SK SR treated with 200 µM H_2O_2, 250 µM NOC-12 and 5 units PKA/ reaction: positive control for calstabin1 dissociation. No antibody in IP: negative control for the RyR1 immunoprecipitation.
g-j Representative immunoblots and quantification of DHPR (**g**, **h**), SERCA-2a (**g**, **i**) and SERCA-1 (**g**, **j**) proteins related to GAPDH protein; n = 8 participants per group. 2-way ANOVA, followed by Sidak's multiple comparisons test (**j**).
k, l Representative immunoblots (**k**) and quantification (**l**) of the ratio of phosphorylated CaMKII on threonine 286 (Thr286) (reported to total proteins) to CaMKII total (reported to total proteins); n = 6 participants per group. 2-way ANOVA.
m-o Representative immunoblots and quantification of NRF1 (**m, n**: n = 8 participants per group) and Tfam (**m, o**: n = 6 participants per group) proteins related to GAPDH protein. 2-way ANOVA.
p-s Representative immunoblots (**p**) and quantification of OPA1 long and short forms (**q, r**: n = 8 participants per group) and DRP1 (**s**: n = 8 participants per group) related to GAPDH protein. 2-way ANOVA.
Data are mean ± SD. *$p \leq 0.05$, **$p \leq 0.01$, $^{\$}$ Main effect of time. Source data are provided as a Source Data file.

Supplementary Fig. 2

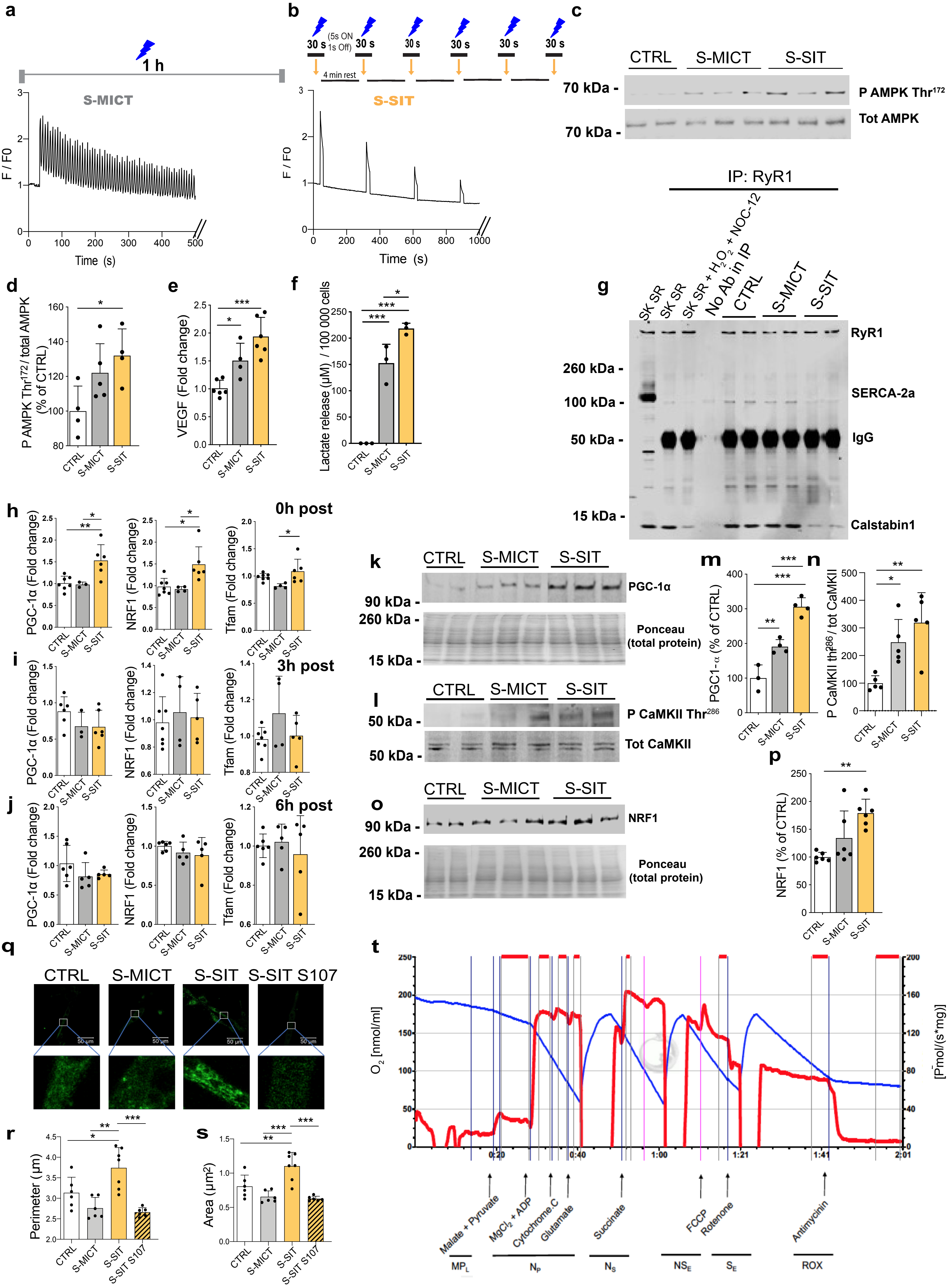

Supplementary Fig. 2. Characterization of in vitro S-MICT and S-SIT models

a, b Normalized Fluo-4 cytosolic fluorescence in response to S-MICT (**a**) and S-SIT (**b**) stimulation.
c, d Representative immunoblots (**c**) and quantification (**d**) of phosphorylated AMPK on threonine 172 and total AMPK immediately post stimulation; n = 4 independent biological experiments per group. 1-way ANOVA followed by Tukey's multiple comparisons test.
e mRNA quantification of VEGF related to Cyclophilin B; n = 6 (CTRL, S-SIT) and 4 (S-MICT) independent biological experiments. 1-way ANOVA followed by Tukey's multiple comparisons test.
f Quantification of lactate release immediately post stimulation; n = 3 independent biological experiments per group. 1-way ANOVA followed by Tukey's multiple comparisons test.
g Immunoblots of non-immunoprecipitated (1st lane) and immunoprecipitated RyR1 samples and immunoblots of RyR1, SERCA-2a and calstabin1. The IgG are shown to distinguish the immunoprecipitated samples from the positive control for SERCA-2a (first lane). SK SR: skeletal muscle sarcoplasmic reticulum vesicle. SK SR treated with 200 μM H_2O_2, 250 μM NOC-12: positive control for calstabin1 dissociation. No antibody in IP: negative control for the RyR1 immunoprecipitation.
h-j mRNA level of PGC-1α, NRF1 and Tfam related to Cyclophilin B immediately (**h**: n = 7 CTRL, 6 S-SIT, 3 S-MICT independent biological experiments), 3h and 6h (**i, j**: n = 7 CTRL, 6 S-SIT, 4 S-MICT independent biological experiments) post stimulation. 1-way ANOVA followed by Tukey's multiple comparisons test (**h**).
k-m Immunoblots (**k**) and quantification (**m**) of PGC-1α proteins related to total protein 72h post stimulation; n = 3 (CTRL) and 4 (S-MICT, S-SIT) independent biological experiments. 1-way ANOVA followed by Tukey's multiple comparisons test.
l-n Representative immunoblots (**l**) and quantification (**n**) of phosphorylated CaMKII on threonine 286 and total CaMKII (both reported to total proteins); n = 5 independent biological experiments per group. 1-way ANOVA followed by Tukey's multiple comparisons test.
o, p Immunoblots (**o**) and quantification (**p**) of NRF1 proteins related to total protein 72h post stimulations; n = 5 independent biological experiments per group. 1-way ANOVA followed by Tukey's multiple comparisons test.
q Mitotracker green fluorescence 72h post stimulation without or with 10 μM S107 treatment.
r, s Quantification of mitochondrial perimeter and area; n = 6 (CTRL, S-MICT, S-SIT S107) and 7 (S-SIT) independent biological experiments. 1-way ANOVA followed by Tukey's multiple comparisons test.
t Original recordings of mitochondrial respiratory capacity in myotubes.
Data are mean ± SD. *$p \leq 0.05$, **$p \leq 0.01$, ***$p \leq 0.001$. Source data are provided as a Source Data file.

Supplementary Fig. 3

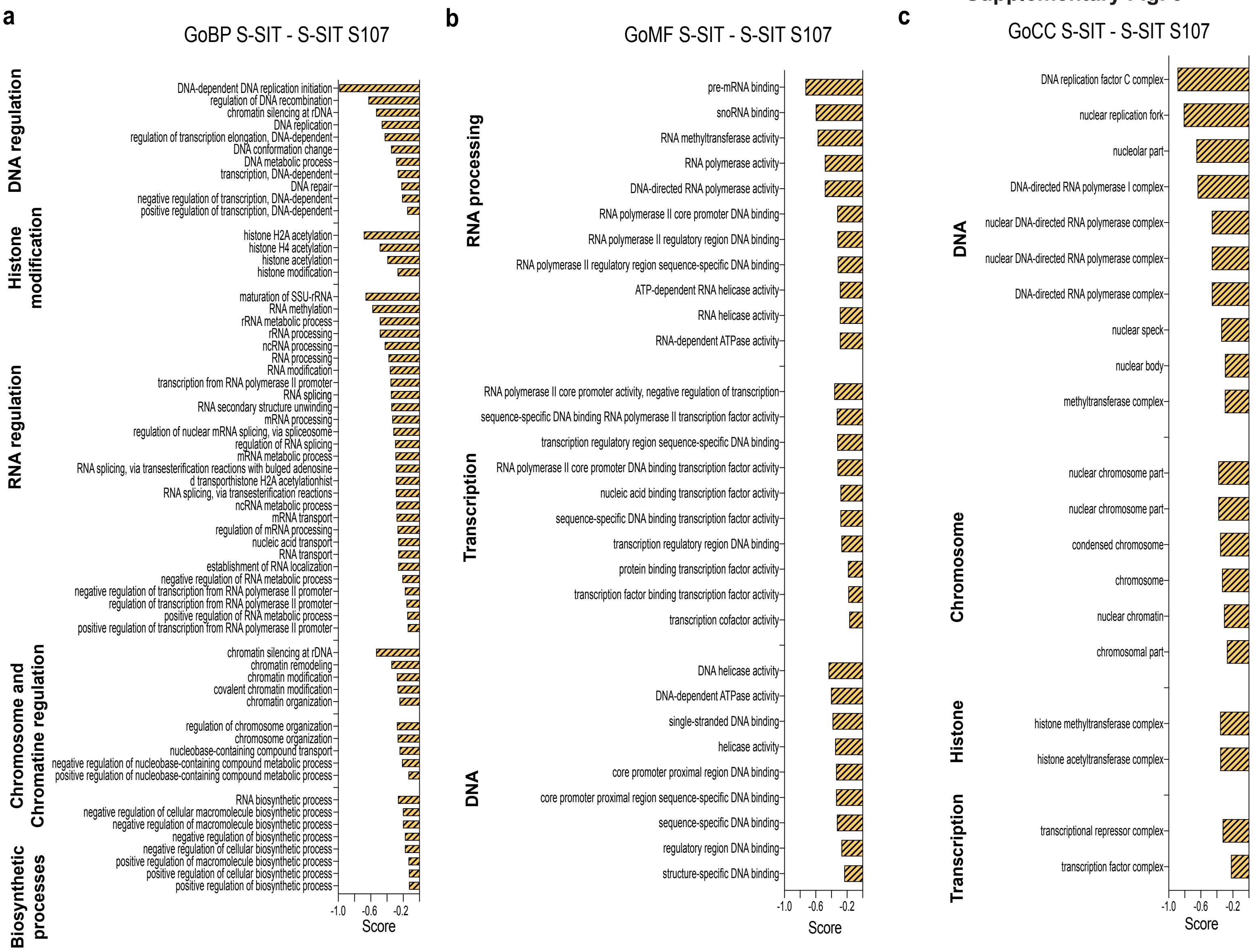

Supplementary Fig. 3. Proteomics data of protein groups increased by S107 treatment in S-SIT myotubes

a-c Proteomic analysis of protein groups related to Gene ontology Biological Processes (GoBP) (**a**), Molecular Function (GoMF) (**b**) and Cellular Component (GoCC) (**c**) that are significantly increased after 72h of 10 μM S107 treatment (the treatment was applied immediately after stimulation). Protein groups exceeding 400 proteins were excluded. The median values of S-SIT – S-SIT S107 difference were calculated, and a score affected to the amplitude of the difference. The negative scores display the pathways significantly increased by S107 treatment. $n = 5$ per group. Benjamini corrected t-test.

Supplementary Fig. 4

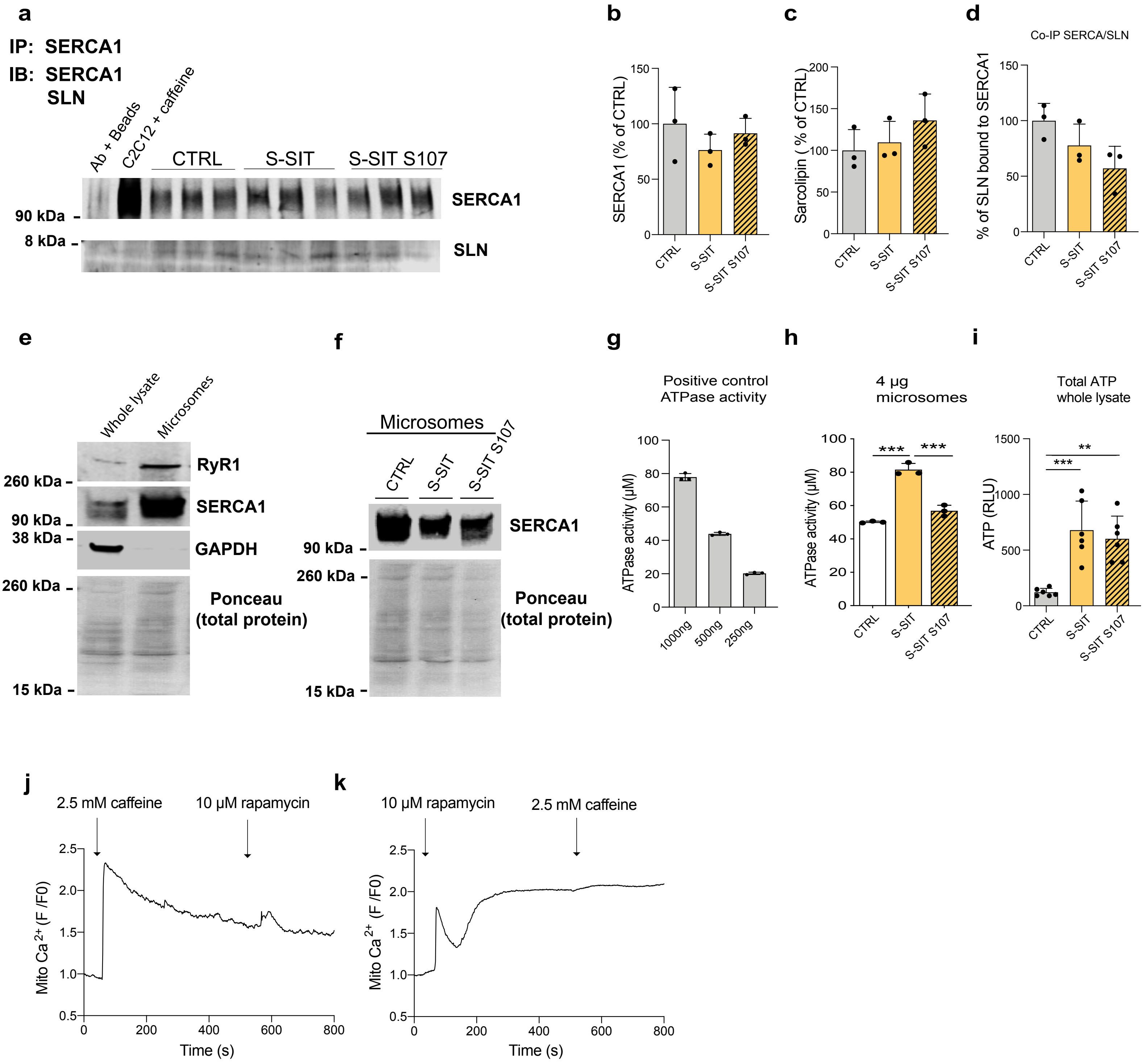

Supplementary Fig. 4. Mitochondrial Ca^{2+} uptake induced by RyR1 opening in C2C12 myotubes and SERCA activity modifications in myotubes in response to S-SIT

a Representative immunoblots of immunoprecipitated SERCA1 and assessment of SERCA1 and sarcolipin (SLN) in myotubes 72h after stimulation without or with 10 µM S107 treatment.
b, d Quantification of SERCA1 (**b**), SLN (**c**) in the input and of the amount of SLN bound to SERCA1 (**d**) in CTRL, S-SIT and S-SIT S107 myotubes; n = 3 independent biological experiments per group. 1-way ANOVA.
e Characterization of microsome preparation. Immunoblot of RyR1, SERCA1 and GAPDH in microsomes (sarcoplasmic reticulum preparation) compared to the whole lysate.
f Representative immunoblots of SERCA-1 in CTRL, S-SIT and S-SIT S107 myotubes (72h after stimulation and treatment).
g Dose-response quantification of ATPase activity in positive control lysate (whole lysate) with 1000, 500 and 250 ng of lysate.
h Quantification of ATPase activity in CTRL, S-SIT and S-SIT S107 myotubes 72h after stimulation and treatment; n = 3 independent biological experiments per group. 1-way ANOVA followed by Tukey's multiple comparisons test.
i Quantification of ATP production in whole cell lysates of CTRL, S-SIT and S-SIT S107 myotubes 72h after stimulation and treatment. RLU (relative light unit); n = 3 independent biological experiments. 1-way ANOVA with Tukey's multiple comparisons test.
j Normalized Rhod-2 fluorescence imaging in C2C12 myotubes showing mitochondrial Ca^{2+} uptake in response to 2.5 mM caffeine stimulation followed by 10 µM rapamycin. Arrows indicate when the treatment was applied.
k Normalized Rhod-2 fluorescence imaging in C2C12 myotubes showing mitochondrial Ca^{2+} uptake in response to 10 µM rapamycin followed by 2.5 mM caffeine application Arrows indicate when the treatment was applied.
The S107 treatment was applied immediately after the stimulation and lasted for 72h
Data are mean ± SD.**$p \leq 0.01$, ***$p \leq 0.001$. Source data are provided as a Source Data file.

Supplementary Fig. 5

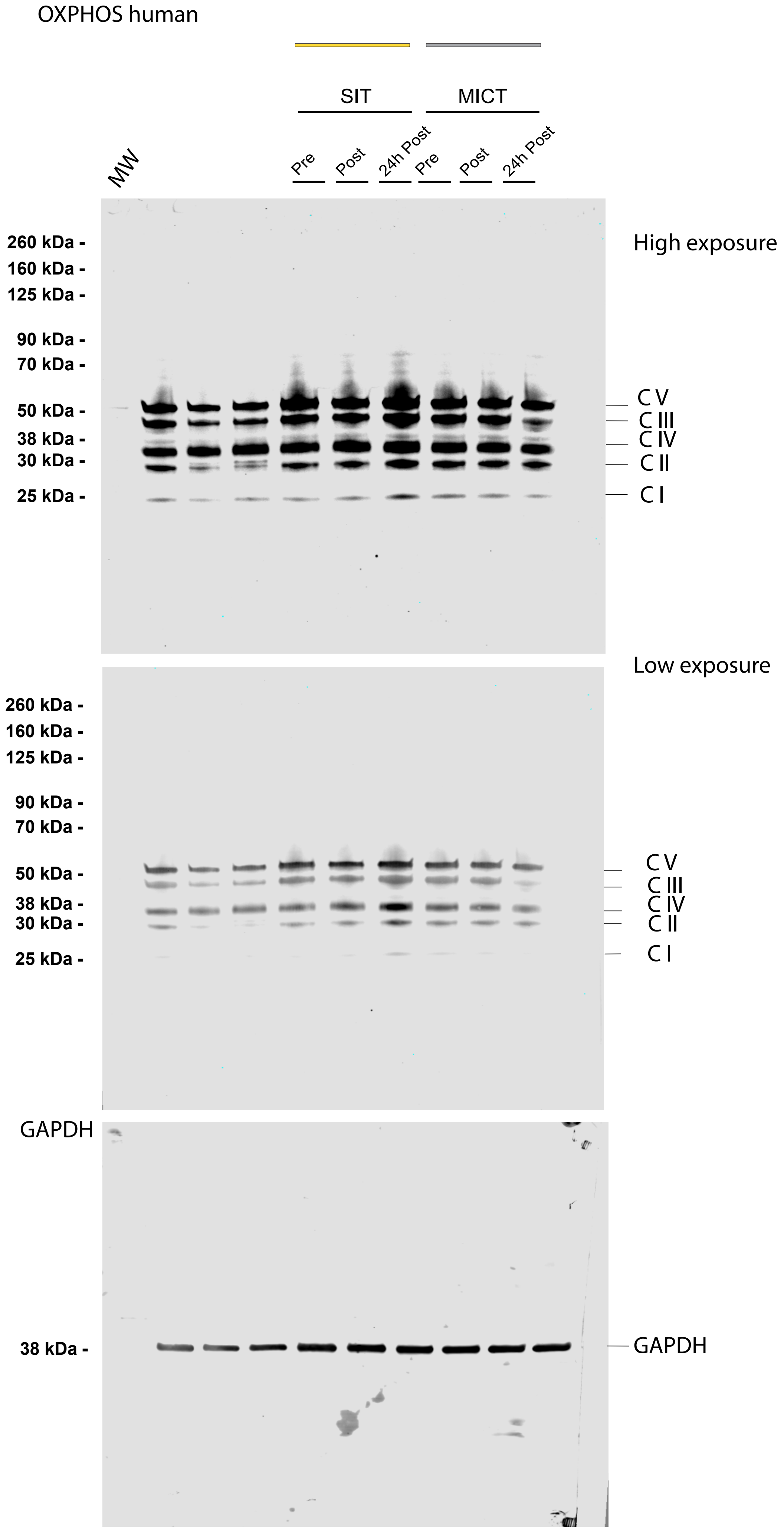

Supplementary Fig. 5. Original gels for human OXPHOS proteins.

Supplementary Fig. 6

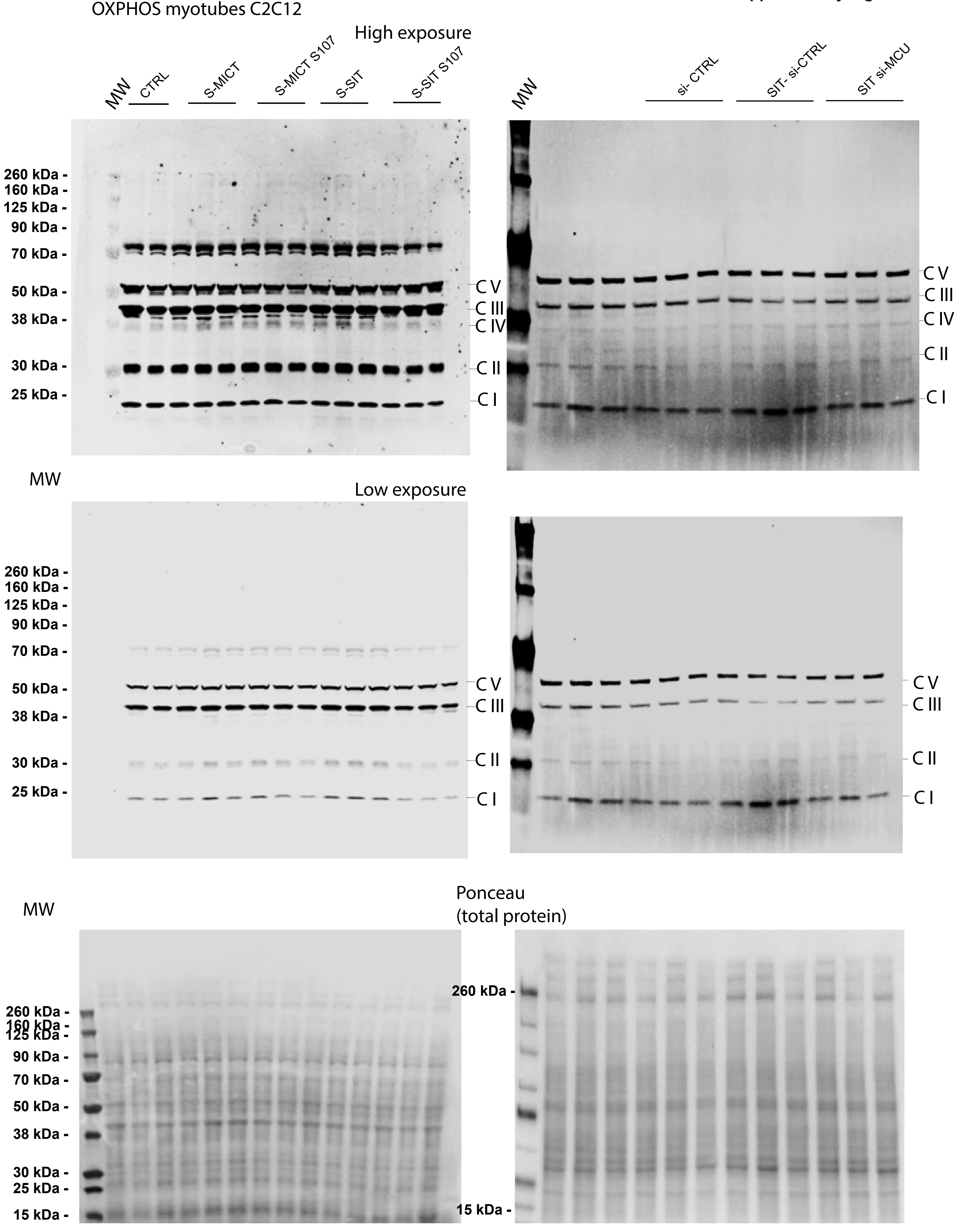

Supplementary Fig. 6. Original gels for C2C12 cell OXPHOS proteins.

Supplementary methods

Ethical Approval

Our research complies with all relevant ethical regulations; the study was validated by the Commission d'éthique de la recherche sur l'être humain du Canton de Vaud (protocol 2017-00303) and by the animal Ethics Committee of Lausanne (commission cantonale pour l'expérimentation animale) with the number VD3489.

Human experiments - general procedures.

The human general procedures were followed as previously described [1] with slight modifications. The study was approved by the local Ethics Committee (protocol 2017-00303) and performed in accordance with the Helsinki declaration. Sixteen male recreationally active subjects gave written informed consent before participation. The participants refrained from physical activity and caffeine consumption for 24 h and 12 h before the experimental session, respectively. Participants were familiarized with electrical stimulation and voluntary contraction procedures at least 48 h before the first experimental session. In the familiarization session they performed an incremental test to exhaustion on a cycle ergometer (Lode Excalibur Sport, Lode, Groningen, Netherlands). The test started at a power of 1 W and was increased by 1 W every 2 s. The participants were instructed to maintain a cadence between 60 and 80 rpm and when they were unable to maintain 60 rpm the test was stopped. The mean of the VO_2 values in the last 30 s of the test was used to determine VO_2peak. Gas exchange was measured with a stationary gas analyzer (Quark CPET, COSMED, Rome, Italy). The participants (n = 8 per group) were allocated in two groups (SIT and MICT) based on their VO_2peak so that mean VO_2peak was similar for each group (Supplementary Fig. 1a).

Moderate intensity continuous training (MICT) consisted of 1 h of cycling on a cycle ergometer at 65% of the maximal aerobic power reached during the incremental VO_2max test. Each experiment was preceded by a standard warm-up on the cycle ergometer (5 min at 100 W). Sprint interval training (SIT) was comprised of 30 s all-out cycling bouts at 0.7 N.m / kg body mass on a cycle ergometer (Lode Excalibur Sport, Lode, Groningen, Netherlands), with 4 min recovery periods (rest) between bouts.

The study was validated by the Commission d'éthique de la recherche sur l'être humain du Canton de Vaud (protocol 2017-00303). Our study complies in full with the STROBE statement.

Analysis of EMG signals.

The electromyographic (EMG) activity of the right *vastus lateralis* was recorded with pairs of silver chloride (Ag/AgCl) circular surface electrodes (1 cm diameter, Kendall Meditrace 100, Tyco, Cork, Ireland) positioned lengthwise over the middle part of the muscle belly with an inter-electrode (center-to-center) distance of 2 cm [2]. The reference electrode was placed over the patella. EMG signals were amplified (gain: 1000), filtered through a 10-500 Hz band-pass filter, and digitized at a sampling frequency of 2 kHz using an AD conversion system (MP150, BIOPAC, Goleta, CA, USA). A high-voltage (maximum 400 V) constant-current stimulator (DS7AH, Digitimer, Hertfordshire, UK) was used to deliver single and paired electrical stimuli (pulse width: 1 ms). The cathode (5 cm diameter, Dermatrode, American Imex, Irvine, CA) and the anode (5 × 10 cm, Compex, Ecublens, Switzerland) were placed over the femoral nerve at the femoral triangle level beneath the inguinal ligament and on the lower part of the gluteal fold opposite to the cathode, respectively. The optimal stimulation intensity was determined by increasing the current until maximal twitch and M-wave amplitude responses were obtained. This intensity was then increased by 20% (i.e. supramaximal) and kept constant for all subsequent tests. Voluntary and evoked forces developed by the knee extensors were recorded at 1 kHz using an isometric ergometer consisting of a custom-built chair equipped with a strain gauge (STS 250 kg, sensitivity 2.0 mV/V and 1.7 mV/N, SWJ, China). The strain gauge was attached to the chair on one end and securely strapped above the ankle with a custom-made mold. Subjects were seated with a knee angle of 90° and a trunk-thigh angle of 100° (180° = full extension). Extraneous movements of the upper body were limited by two crossover shoulder harnesses and a belt across the lower abdomen. Subjects received visual feedback of the force they produced during the MVCs.

Force and EMG data were stored and analysed off-line with commercially available software (AcqKnowledge software, BIOPAC, Goleta, CA, USA). Single electrical stimulation pulses were used to measure the amplitude of the first peak of the M-wave [3] before and after exercise. Isometric MVC force was considered as the peak force attained during the voluntary contraction performed at a given time point. The amplitudes of the 10 Hz and 100 Hz doublets before and after exercise were quantified to assess contractile alterations after exercise. Doublets at 100 Hz were delivered superimposed on and immediately after MVCs to assess the voluntary activation level (VAL), which was used as an index of central fatigue and assessed as: VAL = (1 − (superimposed paired stimuli at 100 Hz × (force level at stimulation/MVC force)/potentiated paired stimuli at 100 Hz)) × 100 [4].

Muscle biopsies

Needle biopsies were taken from the left (non-dominant leg) *vastus lateralis* muscle before, ~10 min and 24 h after exercise, using previously described and validated procedures [5]. Briefly, after skin sterilization and local anesthesia, a 1 to 2 mm long skin cut was made with the tip of a scalpel. Biopsies were collected using an automatic biopsy device (Bard Biopsy Instrument, Bard Radiology, Covington, GA, USA). A 14-gauge disposable trocar mounted in the device was inserted through the cut, perpendicular to the muscle fibres, until the fascia was pierced. Three samples (~15 mg each) were collected from one puncture site at each time point. Muscle samples were immediately frozen in liquid nitrogen and stored at -80°C until analysis.

C2C12 cell culture

C2C12 mouse skeletal myoblasts were obtained from the American Type Culture Collection and grown in proliferation medium composed of Dulbecco's modified Eagle's medium (DMEM; Thermo Fisher Scientific, Basel, Switzerland) supplemented with 10% foetal bovine serum (Thermo Fisher Scientific, Basel, Switzerland), 100 IU/ml penicillin, 100µg/ml streptomycin (Thermo Fisher Scientific, Basel, Switzerland) and 1% non-essential amino acids (Thermo Fisher Scientific, Basel, Switzerland), and maintained at 37 °C in a humidified atmosphere with 5% CO_2. To induce differentiation, myoblasts were grown to 80-90% confluence, the proliferation medium was then replaced with a differentiation medium (DM), consisting of DMEM supplemented with 2% horse serum (Thermo Fisher Scientific, Basel, Switzerland).

Electrical stimulation of C2C12 myotubes

Well differentiated C2C12 myotubes (Day 6 or 7 post differentiation) in 6-well plates (Corning, NY, USA) containing 4 ml of DM were electrically stimulated (C-Pace EM stimulator, IONOPTIX LLC, MA, USA) as follows: 1h repeated stimulation at 14V, 2 Hz and 2 ms pulse duration (S-MICT) or 6 x 30 s pulse (5s on, 1s Off) at 14V, 50 Hz and 2 ms pulse duration with 4 min rest (S-SIT) (Fig. 2a).

The differentiation medium was replaced before and after the electrical stimulation and the cells were harvested at the appropriate time point. Different treatment conditions from the same plate per independent culture were used and the different wells from the same culture were considered as technical replicates. When indicated, 10 µm of S107 (Prof. Marks' lab, USA) was applied to the myotubes after the stimulation for the indicated time.

Cytosolic Ca^{2+} imaging using Fluo-4 AM in C2C12 myotubes

C2C12 myoblasts were plated on Poly-D-Lysin-coated 35-mm-diameter glass-bottom dishes (MatTek, Ashland, MA, USA) adapted for microscopic acquisition. At 80% of confluence, the myoblasts were differentiated by replacing the proliferation medium with DM and myotubes were used at day 5-7 of differentiation. The myotubes were loaded with the cytosolic Ca^{2+} indicator Fluo-4 AM (5µM, Invitrogen, Basel, Switzerland) solubilized in a Krebs solution (in mM: NaCl 135.5, $MgCl_2$ 1.2, KCl 5.9, glucose 11.5, HEPES 11.5, $CaCl_2$ 1.8, final pH 7.3) for 20 min in the incubator. Cells were then rinsed twice with the Krebs solution and myotubes were stimulated using simulated MICT or SIT (S-MICT and S-SIT) protocols and Fluo-4 fluorescence was monitored using a confocal microscope system (Zeiss LSM 5 Live, Oberkochen, Germany; 40x oil immersion lens; the excitation wavelength was 488 nm and the emitted fluorescence was recorded between 495-525 nm). The use of the single excitation/emission Fluo-4 dye necessitated normalizing to pre-stimulation values to account for possible differences in dye loading and excitation strength.

RyR1 immunoprecipitation and immunoblotting

RyR1 immunoprecipitation was performed as previously described [6] with slight modifications. Briefly, muscle biopsies or C2C12 myotubes were isotonically lysed in an ice-cold lysis buffer composed of: 50 mM Tris-HCl (pH 7.4), 150 mM NaCl, 20 mM NaF, 1 mM Na_3VO_4, and protease inhibitors (100 ul per 5 mg muscle tissue or per myotube well of a 6-well plate). The lysates were sonicated then centrifugated at 9'300 g at 4 °C for 10 min. The supernatant was collected, and protein concentration quantified using a BCA assay kit (Thermo Scientific, Basel, Switzerland). An anti-RyR1 antibody (4 µg 5029 Ab, Andrew Marks' lab, Columbia University, NY, USA) was used to immunoprecipitate RyR1 from 250 µg homogenate of human muscle or cells. The samples were incubated with the antibody in 0.5 ml of a modified RIPA buffer (50 mM Tris-HCl pH 7.4, 0.9% NaCl, 5 mM NaF, 1 mM Na_3VO_4, 1% Triton-X100, and protease inhibitors) for 1 hour at 4 °C. The immune complexes were incubated with protein A Sepharose beads (Sigma-Aldrich, St. Louis, MS, USA) at 4°C for 1 hour and the beads were washed three times with buffer. Proteins were separated on SDS-PAGE gels (4-20 % gradient precast gels to visualize both RyR1 and calstabin1 or 6% homemade gels for RyR1 alone and its post-translational modifications) and transferred onto nitrocellulose membranes for 1 hour at 400 mA. After incubation with blocking solution (LICOR Biosciences, Lincoln NE, PBS v/v) to prevent non-specific antibody binding, immunoblots were developed with

rabbit anti-RyR1 (Prof- Marks' lab,1:5000) or rabbit anti-FKBP12 (Abcam, Cambridge, UK, 1:2500) antibodies. IP purity was further tested by investigating SERCA protein contamination (using the rabbit SERCA-2a antibody, Abcam ab137020) as compared to non-IP samples. Sketelal muscle cells were treated with 200 µM H_2O_2 (positive control for RyR oxidation), 250 µM NOC-12 (positive control of RyR nitrosylation) and 5 units PKA / reaction (positive control for RyR phosphorylation). Beads coupled with proteins were used as an IP negative control. Immunoreactive bands were visualized using infrared fluorescence (IR-Odyssey scanner, LI-COR, Lincoln, NE, USA). Band densities were quantified using Image Studio v 5.2.5 (LI-COR, Lincoln, NE, USA).

Western blot analysis

A lysis buffer containing the following: 20 mM Tris/HCl (pH 6.8), 2 mM EDTA (pH 8), 137 mM NaCl, 10% glycerol, 10% Triton X-100, 10 mM glycero-phosphate, 1 mM KH_2PO_4, 1 mM PMSF, 1 mM $NaVO_3$, 50 mM NaF, 10 mM NaPPi, and a protease inhibitor mixture (Roche, Complete Mini, Basel, Switzerland) was used to resuspend myotube pellets (100 ul / well of a 6-well plate) or human muscle samples (100 ul / 5 mg of tissue). The preparation was homogenized with pipette tips for cells or potter for muscles, incubated 1 h at 4 °C and gently sonicated. Then, nuclei and debris were removed by centrifugation at 10'000 *g* at 4 °C for 10 min. Protein quantification was assessed using the BCA kit (Thermo Fisher Scientific, Ecublens, Switzerland). Fifteen to 20 ug of protein were incubated with 2 x Laemmli sample buffer containing SDS and 2-mercapto-ethanol (Bio-Rad, Hercules, CA, USA) for 3 min at 95 °C, electrophoresed 1 h or 2 h (for OPA1 protein) on 4-15% SDS-precast gradient gels (Bio-Rad, Hercules, CA, USA), and wet transferred 1 h onto PVDF membranes. Membranes were stained with Red Ponceau (homemade) and total protein bands were quantified using the Image Studio software v 5.2.5 (LI-COR, Lincoln, NE, USA). Then, the Red Ponceau was washed out with PBS and the membranes were saturated 1h at room temperature with PBS-LI-COR blocking buffer (LI-COR, Lincoln, NE, USA). Blots were incubated overnight with mouse anti-DHPR (Abcam, Cambridge, UK, 1:1000), rabbit anti-FKBP12 (Abcam, Cambridge, UK, 1:2500), mouse anti-SERCA-2a (Abcam, Cambridge, UK, 1:1000), rabbit anti-SERCA1 (Abcam, Cambridge, UK, 1:1000), rabbit anti-sarcolipin (SLN) (Sigma, St Louis, USA, 1:1000), rabbit anti-DRP1 (Abcam, Cambridge, UK, 1:1000), mouse anti-PGC1-α (Sigma, St Louis, USA, 1:1000), rabbit anti-TFAM (Abcam, Cambridge, UK, 1:1000), rabbit anti-NRF1

(Abcam, Cambridge, UK, 1:1000), mouse anti-OPA1 (BD Biosciences, San Jose, US, 1:1000), rabbit anti-GAPDH (Sigma, St Louis, USA, 1:5000), rabbit anti-AMPKα phospho threonine 172 (Cell Signaling, Leiden, The Netherlands, 1:1000), mouse anti-AMPK total (Cell signaling, Leiden, The Netherlands, 1:1000), rabbit CaMKII phospho threonine 286 (Cell signaling, Leiden, The Netherlands, 1:1000), rabbit CaMKII total (Cell signaling, Leiden, The Netherlands, 1:1000). Membranes were washed in PBS-buffered saline-Tween 20 (TBS-T) and incubated for 1 h at room temperature with IRDye 680- conjugated donkey anti-mouse or rabbit IgG (LI-COR, Lincoln, NE, USA, 1:10000) and IRDye 800-conjugated donkey anti-mouse or rabbit IgG (LI-COR, Lincoln, NE, USA, 1: 5000) in blocking buffer. Immunoreactive bands were visualized using infrared fluorescence (IR-Odyssey scanner, LI-COR, Lincoln, NE, USA). Band densities were quantified using Image Studio v 5.2.5 (LI-COR, Lincoln, NE, USA). Protein intensity signal was normalized to that of GAPDH (which was stable across samples and conditions) in human samples while total protein staining (found as a more representative loading control for cells) was used to normalize protein content quantified in cells. The LiCor system allows detection of different proteins at the same time (revealed in different channels). When needed, the membranes were stripped using the appropriate LiCor stripping solution (LiCor, Lincoln, NE, USA). For human samples, all the protein quantifications were expressed as a percentage of the Pre MICT values (kept as the reference for ANOVA testing). The protein quantifications for the cell samples were reported to that of CTRL.

SERCA-SLN co-immunoprecipitation

Myotubes were lysed in a lysis buffer containing the following: 20 mM Tris/HCl (pH 6.8), 2 mM EDTA (pH 8), 137 mM NaCl, 1% NP40, 10 mM glycero-phosphate, 1 mM KH_2PO_4, 1 mM PMSF, 1 mM $NaVO_3$, 50 mM NaF, 10 mM NaPPi, and a protease inhibitor mixture (Roche, Complete Mini, Basel, Switzerland). The final volume used was 100 µl per well of a 6-well plate. The preparation was homogenized with pipette tips, and incubated for 1 h at 4 °C. Then, nuclei and debris were removed by gentle centrifugation at 300 g at 4 °C for 5 min. Protein quantification was assessed using the BCA kit (Thermo Fisher Scientific, Ecublens, Switzerland). 40 µl of Dynabead protein G (Thermo Fisher Scientific, Ecublens, Switzerland) were coupled to 2 µg of SERCA1 antibody (Abcam, Cambridge, UK) in 0.5 ml of the lysis buffer for 2 h at 4°C. The complexes were washed 3 times with the lysis buffer, and then

combined to 250 μg of cell homogenate overnight at 4°C. The immune complexes were washed 3 times, then diluted with 20 μl of 2 x Laemmli sample buffer containing SDS and 2-mercapto-ethanol (Bio-Rad, Hercules, CA, USA). The samples were heated 3 min at 75 °C then 3 min at 95 °C, centrifuged at 300 g for 30 s. Proteins were separated on 4-15% SDS-precast gradient gels (Bio-Rad, Hercules, CA, USA), and wet transferred for 1 h onto PVDF membranes. The membranes were blocked then successively blotted with 1:1000 rabbit anti-SERCA1 antibody (Abcam, Cambridge, UK) and 1:1000 rabbit anti-SLN antibody (Sigma, St Louis, USA). Myotubes treated 15 min with 2.5 mM of caffeine were used as positive controls and antibody/beads complex used as negative controls. The band intensities were determined as described above and the SLN/SERCA1 ratio was calculated. The input levels of SERCA1 and SLN proteins in the lysates were assessed by western blot.

SR Vesicle (microsome) preparation

SR vesicles from C2C12 myotubes were prepared by lysing on ice cell pellets with 1 ml/ mg of a homogenizing buffer composed of 10 mM Tris-maleate, 1 mM EDTA and protease inhibitors (Roche) (final pH adjusted to 7.4). The homogenate was briefly sonicated and then centrifuged at 8'000 g for 20 min at 4°C. The resulting supernatant was centrifuged at 50'000 g for 1h at 4°C. The final pellet, containing the SR fractions, was collected in the resuspending buffer composed of 10 mM Tris-maleate buffer, 250 mM sucrose, 10 mM MOPS, 1 mM EDTA, and protease inhibitors (pH adjusted to 7.4). The final volume was 50 μl of buffer per 3mg of initial tissue. Protein quantification was assessed using the BCA kit (Thermo Fisher Scientific, Ecublens, Switzerland) and the samples were frozen in liquid nitrogen and stored at -80°C until analysis. A quality control of the microsomes was performed by checking enrichment of RyR1 and SERCA1 proteins in the microsome fractions compared to a whole cell lysate and exclusion of cytosolic proteins such as GAPDH (Supplementary Fig. 4g).

Ca^{2+} ATPase measurements

Ca^{2+} ATPase activity was measured using 4 μg of the microsome fraction from each condition using a colorimetric ATPase assay kit following the manufacturer's instructions (Sigma, St. Louis, USA). A dose response assay was performed on a whole cell lysate used as a positive control (Supplementary Fig. 4i).

Real-time polymerase chain reaction

Myotubes were homogenized in TRIzol (Invitrogen, Basel, Switzerland) at a concentration of 500 µl per well of a 6-well plate and the extracted RNA was quantified using the Nanodrop (Thermo Fisher Scientific, Basel, Switzerland). 1 µg RNA was reverse transcribed using qScript Reverse Transcriptase (Invitrogen, Basel, Switzerland). Cyclophilin B housekeeping gene and genes of interest were amplified in parallel. RT-PCR was performed using 2 µl of cDNA, 9 µl of GoTaq qPCR Master Mix (Promega, Dübendorf, Switzerland) and 400 nM of each primer in a total reaction volume of 11 µl. Data were recorded on a ViiA7 real-time RT-PCR detection system (Life Technologies, Basel, Switzerland) and cycle threshold (Ct) values for each reaction were determined using analytical software QuantStudio 2016 version from the same manufacturer. Each cDNA was amplified in triplicate, and Ct values were averaged for each triplicate. The average Ct value for Cyclophilin B was subtracted from the average Ct value for the gene of interest and normalized to control cells. As amplification efficiencies of the genes of interest and Cyclophilin B were comparable, the amount of mRNA, normalized to Cyclophilin B was given by the relation $2^{-\Delta\Delta Ct}$. The used primers were designed as previously reported for [7,8] and primer sequences are reported in the supplementary data file 3.

Measurement of mitochondrial morphology in C2C12 cells

Myotubes were incubated in 200 nM Mitotracker green (Invitrogen, Basel, Switzerland) in Krebs solution (mM: NaCl 135.5, $MgCl_2$ 1.2, KCl 5.9, glucose 11.5, HEPES 11.5, $CaCl_2$ 1.8, final pH 7.3) and protected from light for 15 min at 37°C. Rhod-2 florescence was detected by using a confocal laser scanning microscopy (inverted Zeiss LSM 710 confocal microscope, Oberkochen, Germany) technique to define single layers of cells at 40x magnification. Mitochondrial shape descriptors and size measurement were determined using Fiji [9], an enhanced version of ImageJ Software (http://fiji.sc/) as previously described [10,11].

Lactate release assay

Lactate release assay was performed on C2C12 myotubes immediately after electrical stimulation. About 100,000 myoblasts were plated per well of a 6-well plate and differentiated until day 7. The DM was changed before the electrical stimulation. Immediately after MICT or SIT stimulation, the culture medium was collected, and lactate concentration was assessed as

previously reported [12]. 200 µl of the culture medium per well was mixed with a 0.33 M glycine-semicarbazide buffer supplemented with 0.015 M nicotinamide adenine dinucleotide (Roche, Basel, Switzerland) and 70 U/ml L-lactate dehydrogenase (Roche, Basel, Switzerland). The quantification was performed by measuring the NADH produced via the L-lactate dehydrogenase-catalyzed enzymatic reaction with a microplate reader (SynergyMx; BioTek, Luzern, Switzerland) at 340 nm. Lactate values were normalized to the number of plated cells per well and expressed as nM / 100,000 cells and compared to the lactate level immediately before stimulation (control condition).

Proteomics data acquisition

Protein digestion

Differentiated C2C12 myotubes were exposed to the SIT stimulation protocol and then divided in two groups with one immediately treated after the SIT session with 10 µM S107 for 72 h. After 72 h, cell pellets were collected and stored at -80 °C for later analysis. Replicate samples of SIT and SIT S107 (5 per group) were digested with the miST method (modified version of the in-StageTip method, [13]). Briefly, frozen cell pellets were resuspended in 100 µl miST lysis buffer (1% sodium deoxycholate, 100 mM Tris pH 8.6, 10 mM DTT) by vigorous vortexing. Resuspended samples were heated at 95 °C for 5 min, and 100 µg of protein were transferred into new tubes, based on tryptophane quantification [14]. Samples were then diluted 1:1 (v:v) with water containing 4 mM $MgCl_2$ and benzonase (Merck #70746, 100x dilution of stock = 250 units/µl) and incubated for 15 min at room temperature to digest nucleic acids. Reduced disulfides were alkylated by adding ¼ vol (25 µl) of 160 mM chloroacetamide (final 32 mM) and incubating at 25°C for 45 min in the dark. Samples were adjusted to 3 mM EDTA and digested with 1 µg Trypsin/LysC mix (Promega #V5073) for 1 h at 37 °C, followed by a second 1 h digestion with a second and identical aliquot of proteases. To remove sodium deoxycholate, two sample volumes of isopropanol containing 1% Trifluoroacetic acid (TFA) were added to the digests, and the samples were desalted on a strong cation exchange (SCX) plate (Oasis MCX; Waters Corp., Milford, MA, USA) by centrifugation. After washing with isopropanol/1%TFA, peptides were eluted in 250 µl of 80% MeCN, 19% water, 1% (v/v) ammonia.

Tandem Mass Tag (TMT) labelling

Eluates after SCX desalting were dried and resuspended in 100 µl water. 30 µl of digests were then aliquoted and dried again, before resuspension in 25 µl of 50 mM TEAB buffer, pH 8.0. For labelling, 0.2 mg of TMT reagent in 20 µl acetonitrile were added to the samples for 1h at room temperature, after which excess reagent was quenched with 1 µl of 5% hydroxylamine for 15 min at room temperature.

An aliquot (0.8 µl) was injected before mixing to assess labelling completion (> 98% peptide spectrum matches) by database search with TMT as variable modification (MASCOT software, www.matrixscience.com). After mixing, the TMT multiplex sample was dried and desalted on a SepPak micro C18 96-well plate (Waters Corp., Milford, MA, USA).

Peptide fractionation

The dried desalted eluate was dissolved in 4 M urea containing 0.1% ampholytes pH 3-10 (GE Healthcare, Ecublens, Switzerland). Then 75% of the sample (225 µg) was fractionated by off-gel focusing as described [15]. The 24 peptide fractions obtained were desalted on a SepPak micro C18 96-well plate, dried and dissolved in 25 µl of 0.05% trifluroacetic acid, 2% (v/v) acetonitrile for liquid chromatography-mass spectrometry/ mass spectrometry (LC-MS/MS) analysis.

MS analysis

Data-dependent LC-MS/MS analysis of TMT samples was carried out on a Fusion Tribrid Orbitrap mass spectrometer (Thermo Fisher Scientific, Basel, Switzerland) interfaced through a nano-electrospray ion source to an Ultimate 3000 RSLCnano HPLC system (Dionex, Thermo Fisher Scientific, Basel, Switzerland). Peptides were separated on a reversed-phase custom packed 40 cm C18 column (75 µm ID, 100Å, Reprosil Pur 1.9 µm particles, Dr. Maisch HPLC Gmbh, Ammerbuch-Entringen, Germany) with a 4-76% acetonitrile gradient in 0.1% formic acid (total time = 140 min). Full MS survey scans were performed at 120’000 resolution. A data-dependent acquisition method controlled by Xcalibur 4.2 software (Thermo Fisher Scientific, Basel, Switzerland) was used to optimize the number of precursors selected (“top speed”) of charge 2^+ to 5^+ while maintaining a fixed scan cycle of 1.5 s. The precursor isolation window used was 0.7 Th.

Peptides were fragmented by higher energy collision dissociation with a normalized energy of 37%. MS2 scans were performed at a resolution of 50,000 in the Orbitrap cell, to resolve 10-

plex TMT reporter ions. The *m/z* of fragmented precursors was then dynamically excluded from selection during 60 s.

MS data analysis

Data files were analysed with MaxQuant 1.6.3.4 [16,17], incorporating the Andromeda search engine [17]. Cysteine carbamidomethylation and TMT labelling (peptide N-termini and Lysine side chains) were selected as fixed modifications while methionine oxidation and protein N-terminal acetylation were specified as variable modifications. The sequence databases used for searching were the mouse (*Mus musculus*) Reference Proteome based on the UniProt database (www.uniprot.org, version of January 31th, 2019, containing 54,211 sequences), and a "contaminant" database containing the most usual environmental contaminants and the enzymes used for digestion (keratins, trypsin, etc). Mass tolerance was 4.5 ppm on precursors (after recalibration) and 20 ppm on higher energy collision dissociation fragments. Both peptide and protein identifications were filtered at 1% FDR relative to hits against a decoy database built by reversing protein sequences. For TMT analysis, the raw reporter ion intensities generated by MaxQuant (with a mass tolerance of 0.003 Da) and summed for each protein group were used in all following steps to derive quantitation.

Processing of quantitative data and statistical tests

The MaxQuant output table "proteinGroups.txt" was processed with Perseus software [18] to remove proteins matched to the contaminants database as well as proteins identified only by modified peptides or reverse database hits, and those without any quantitative values, yielding a first unfiltered list of 7229 identified proteins. Next, the table was filtered to retain only proteins identified by a minimum of three peptides (5870 protein groups left).

After log-2 transformation of all intensity values, and normalization by median substraction, a two-samples t-test with Benjamini-Hochberg FDR correction (threshold at 0.05 on the adjusted p value; [19] was performed between the SIT and SIT S107 groups and showed 365 significant proteins. Gene ontology annotation enrichment test was carried out with Perseus, using average difference in log-2 scale between both groups, and applying a Benjamini-Hochberg FDR of 2%. The resulting score used in Fig. 4 and Supplementary Fig. 3 indicates how far is the center of the distribution of values for the protein category considered relatively to the overall distribution of values [20]. The interval of this positional score is between -1 and 1.

Raw data deposition

All the mass spectrometry proteomics raw data together with MaxQuant output tables are available via the Proteomexchange Consortium via the PRIDE [21] partner repository with the dataset identifier PXD018409.

Submission details:

Project Name: "Triggering acute RyR1 Ca^{2+} leak leads to improved mitochondrial remodelling and function"

Project accession: PXD018409
Project DOI: Not applicable

The data are accessible on the PRIDE web site: http://www.proteomexchange.org
under the accession number: PXD018409
[http://www.ebi.ac.uk/pride/archive/projects/PXD018409]

Analysed proteomics data are provided in Supplementary data 1 and 2. SIE (sprint interval exercise) refers to SIT.

Statistical analysis

Except for proteomic analyses, data are presented as mean ± SD. When data were normally distributed, analysis of variance (ANOVA) was performed to compare many groups. Two-way ANOVA was followed by Tukey's or Sidak's multiple comparisons post hoc tests to compare many groups or multiple time points when the interaction was significant. One-way ANOVA was followed by Tukey's or Sidak's multiple comparisons post hoc tests to compare different groups when the main effect was significant. When data were not normally distributed, Kruskal–Wallis ANOVA test followed by Dunn's multiple comparisons were used to compare different groups. Data were analysed using GraphPad Prism version 8.4.2 and SigmaPlot version 11.0. The level of significance was fixed at $p \leq 0.05$. All detailed p values are presented in Supplementary Data 5. The figures were mounted using Adobe Illustrator 23.0.3.

Supplementary references

1 Place, N. *et al.* Ryanodine receptor fragmentation and sarcoplasmic reticulum Ca2+ leak after one session of high-intensity interval exercise. *Proc Natl Acad Sci U S A* **112**, 15492-15497, doi:10.1073/pnas.1507176112 (2015).

2 Hermens, H. J., Freriks, B., Disselhorst-Klug, C. & Rau, G. Development of recommendations for SEMG sensors and sensor placement procedures. *J Electromyogr Kinesiol* **10**, 361-374, doi:10.1016/s1050-6411(00)00027-4 (2000).
3 Rodriguez-Falces, J. & Place, N. Determinants, analysis and interpretation of the muscle compound action potential (M wave) in humans: implications for the study of muscle fatigue. *Eur J Appl Physiol* **118**, 501-521, doi:10.1007/s00421-017-3788-5 (2018).
4 Strojnik, V. & Komi, P. V. Neuromuscular fatigue after maximal stretch-shortening cycle exercise. *J Appl Physiol (1985)* **84**, 344-350, doi:10.1152/jappl.1998.84.1.344 (1998).
5 Magistris, M. R. *et al.* Needle muscle biopsy in the investigation of neuromuscular disorders. *Muscle Nerve* **21**, 194-200, doi:10.1002/(sici)1097-4598(199802)21:2<194::aid-mus6>3.0.co;2-6 (1998).
6 Andersson, D. C. *et al.* Ryanodine receptor oxidation causes intracellular calcium leak and muscle weakness in aging. *Cell Metab* **14**, 196-207, doi:10.1016/j.cmet.2011.05.014 (2011).
7 Peters, E. L. *et al.* IGF-1 Attenuates Hypoxia-Induced Atrophy but Inhibits Myoglobin Expression in C2C12 Skeletal Muscle Myotubes. *Int J Mol Sci* **18**, doi:10.3390/ijms18091889 (2017).
8 Burch, N. *et al.* Electric pulse stimulation of cultured murine muscle cells reproduces gene expression changes of trained mouse muscle. *PLoS One* **5**, e10970, doi:10.1371/journal.pone.0010970 (2010).
9 Schindelin, J. *et al.* Fiji: an open-source platform for biological-image analysis. *Nat Methods* **9**, 676-682, doi:10.1038/nmeth.2019 (2012).
10 Burbulla, L. F. & Kruger, R. The use of primary human fibroblasts for monitoring mitochondrial phenotypes in the field of Parkinson's disease. *J Vis Exp*, doi:10.3791/4228 (2012).
11 Picard, M. *et al.* Mechanical ventilation triggers abnormal mitochondrial dynamics and morphology in the diaphragm. *J Appl Physiol (1985)* **118**, 1161-1171, doi:10.1152/japplphysiol.00873.2014 (2015).
12 Rosenberg, J. C. & Rush, B. F. An enzymatic-spectrophotometric determination of pyruvic and lactic acid in blood. Methodologic aspects. *Clin Chem* **12**, 299-307 (1966).
13 Kulak, N. A., Pichler, G., Paron, I., Nagaraj, N. & Mann, M. Minimal, encapsulated proteomic-sample processing applied to copy-number estimation in eukaryotic cells. *Nat Methods* **11**, 319-324, doi:10.1038/nmeth.2834 (2014).
14 Wisniewski, J. R. & Gaugaz, F. Z. Fast and sensitive total protein and Peptide assays for proteomic analysis. *Anal Chem* **87**, 4110-4116, doi:10.1021/ac504689z (2015).
15 Geiser, L., Dayon, L., Vaezzadeh, A. R. & Hochstrasser, D. F. Shotgun proteomics: a relative quantitative approach using Off-Gel electrophoresis and LC-MS/MS. *Methods Mol Biol* **681**, 459-472, doi:10.1007/978-1-60761-913-0_27 (2011).
16 Cox, J. & Mann, M. MaxQuant enables high peptide identification rates, individualized p.p.b.-range mass accuracies and proteome-wide protein quantification. *Nat Biotechnol* **26**, 1367-1372, doi:10.1038/nbt.1511 (2008).
17 Cox, J. & Mann, M. Quantitative, high-resolution proteomics for data-driven systems biology. *Annu Rev Biochem* **80**, 273-299, doi:10.1146/annurev-biochem-061308-093216 (2011).
18 Tyanova, S., Temu, T. & Cox, J. The MaxQuant computational platform for mass spectrometry-based shotgun proteomics. *Nat Protoc* **11**, 2301-2319, doi:10.1038/nprot.2016.136 (2016).

19 Hochberg, Y. & Benjamini, Y. More powerful procedures for multiple significance testing. *Stat Med* **9**, 811-818, doi:10.1002/sim.4780090710 (1990).
20 Cox, J. & Mann, M. 1D and 2D annotation enrichment: a statistical method integrating quantitative proteomics with complementary high-throughput data. *BMC Bioinformatics* **13 Suppl 16**, S12, doi:10.1186/1471-2105-13-S16-S12 (2012).
21 Perez-Riverol, Y. *et al.* The PRIDE database and related tools and resources in 2019: improving support for quantification data. *Nucleic Acids Res* **47**, D442-D450, doi:10.1093/nar/gky1106 (2019).
