# Supplementary material for: Acute RyR1 Ca2+ leak enhances NADH-linked mitochondrial respiratory capacity
Source: Nat Commun. 2021 Dec 10;12:7219. doi: 10.1038/s41467-021-27422-1 (PMC8664928; doi:10.1038/s41467-021-27422-1)
Supplement: Supplementary file 3 — Description of Additional Supplementary Files [file 41467_2021_27422_MOESM3_ESM.pdf]

File name: Supplementary Data 1

Description: **Proteins identified by the proteomic analysis of SIT and SIT S107 myotubes**. Gene ontology Biological Processes (GoBP), Molecular Function (GoMF) and Cellular Component (GoCC) of all individual proteins identified by the proteomic analysis of S-SIT and S-SIT S107 myotubes; the S107 treatment was applied immediately after S-SIT stimulation and lasted for 72h.

File name: Supplementary Data 2

Description: **Protein group enrichment of SIT and SIT S107 myotubes.** Gene ontology Biological Processes (GoBP), Molecular Function (GoMF) and Cellular Component (GoCC) of all protein groups statistically different between S-SIT and S-SIT S107 myotubes. The statistics are based on S-SIT – S-SIT S107 median difference. Positive scores indicate all protein groups inhibited by 10 μM S107 treatment and negative scores indicate all the protein groups increased by 10 μM S107 treatment; the S107 treatment was applied immediately after S-SIT stimulation and lasted for 72h.

File name: Supplementary Data 3

Description: **Detailed information on the antibodies, reagents, primers et software used in this study**

File name: Supplementary Data 4

Description: **Detailed metabolite quantification database**

File name: Supplementary Data 5

Description: **Detailed statistical data (main, interaction and post hoc tests p values)**
