# Supplementary material for: Acute RyR1 Ca2+ leak enhances NADH-linked mitochondrial respiratory capacity
Source: Nat Commun. 2021 Dec 10;12:7219. doi: 10.1038/s41467-021-27422-1 (PMC8664928; doi:10.1038/s41467-021-27422-1)
Supplement: Supplementary file 9 — Reporting Summary [file 41467_2021_27422_MOESM9_ESM.pdf]

nature research

Corresponding author(s): Nadège Zanou

Last updated by author(s): Oct 23, 2021

Reporting Summary

Nature Research wishes to improve the reproducibility of the work that we publish. This form provides structure for consistency and transparency in reporting. For further information on Nature Research policies, see our Editorial Policies and the Editorial Policy Checklist.

Statistics

For all statistical analyses, confirm that the following items are present in the figure legend, table legend, main text, or Methods section.

n/a	Confirmed	
☐	☒	The exact sample size (*n*) for each experimental group/condition, given as a discrete number and unit of measurement
☐	☒	A statement on whether measurements were taken from distinct samples or whether the same sample was measured repeatedly
☐	☒	The statistical test(s) used AND whether they are one- or two-sided<br>*Only common tests should be described solely by name; describe more complex techniques in the Methods section.*
☒	☐	A description of all covariates tested
☐	☒	A description of any assumptions or corrections, such as tests of normality and adjustment for multiple comparisons
☐	☒	A full description of the statistical parameters including central tendency (e.g. means) or other basic estimates (e.g. regression coefficient) AND variation (e.g. standard deviation) or associated estimates of uncertainty (e.g. confidence intervals)
☒	☐	For null hypothesis testing, the test statistic (e.g. *F*, *t*, *r*) with confidence intervals, effect sizes, degrees of freedom and *P* value noted<br>*Give P values as exact values whenever suitable.*
☒	☐	For Bayesian analysis, information on the choice of priors and Markov chain Monte Carlo settings
☒	☐	For hierarchical and complex designs, identification of the appropriate level for tests and full reporting of outcomes
☒	☐	Estimates of effect sizes (e.g. Cohen's *d*, Pearson's *r*), indicating how they were calculated

Our web collection on statistics for biologists contains articles on many of the points above.

Software and code

Policy information about availability of computer code

Data collection	Force and EMG data were stored and analyzed off-line with commercially available software (AcqKnowledge 4.2 software, BIOPAC, Goleta, CA, USA).<br>Zen software 2012 version (Zeiss, Oberkochen, Germany) was used for the acquisition of Cytosolic and mitochondrial Ca2+ imaging.<br>Mitochondrial respiration rates were assessed using a slightly modified SUIT-008 protocol of the DatLab 7.3 software (Oroboros, Innsbruck, Austria).<br>Via7 real-time RT-qPCR detection system (Life Technologies, Basel, Switzerland) and the related analytical software QuantStudio real time PCR software 2016 version (Life Technologies, Basel, Switzerland) were used to determine the cycle threshold (Ct) values for each reaction.<br>Xcalibur 4.2 software (Thermo Fisher Scientific, Basel, Switzerland) was used to optimize the number of precursors selected in the LC-MS technique.<br>Mascot software 2.6 (www.matrixscience.com) was used to identify the TMT peptide spectrum with in the mass spectrometry.<br>For western blot, immunoreactive bands were visualized using infrared fluorescence (IR-Odyssey scanner, LICOR, Lincoln, NE, USA) and the related software Image Studio v 5.2.5 (LI-COR, Lincoln, NE, USA).
Data analysis	Total protein bands were quantified using the Image Studio software v 5.2.5 (LI-COR, Lincoln, NE, USA).<br>Mitochondrial shape descriptors and size measurement were determined using Fiji 2.0, an enhanced version of ImageJ Software (http://fiji.sc/)<br>For SR Ca2+ measurements, fluorescence ratios were calculated in MetaFluor 7.0 (Meta Imaging Series, Molecular Devices, San Jose, CA, USA).<br>Via7 real-time RT-qPCR detection data were further analysed with Excel.<br>LI-COR immunoreactive band densities were quantified using Image Studio 5.2.5 (LI-COR, Lincoln, NE, USA).<br>All data analysis was finalized using Excel (Microsoft, Seattle, WA, USA) and GraphPad Prism 8.3.1 (GraphPad, San Diego, CA, USA).<br>Raw LC-MS/MS data was processed using the Agilent Quantitative analysis software B.07.00 (MassHunter Agilent technologies).<br>The MaxQuant output table "proteinGroups.txt" was processed with Perseus software (https://maxquant.net/perseus/)<br>Adobe Illustator 23.0.3 software was used to prepare all the figures presented in this paper.

BioRender application (https://app.biorender.com) was used design schematics.

For manuscripts utilizing custom algorithms or software that are central to the research but not yet described in published literature, software must be made available to editors and reviewers. We strongly encourage code deposition in a community repository (e.g. GitHub). See the Nature Research guidelines for submitting code & software for further information.

Data

Policy information about availability of data

All manuscripts must include a data availability statement. This statement should provide the following information, where applicable:

- Accession codes, unique identifiers, or web links for publicly available datasets
- A list of figures that have associated raw data
- A description of any restrictions on data availability

All data generated and analysed during this study are included in this published article (and its supplementary information files).
The proteomics data generated in this study have been deposited in the Pride website (http://www.proteomexchange.org) under accession code PXD018409. Analysed proteomics data are provided in Supplementary data 1 and 2. The data related to the metabolite quantification of this study are presented in the supplementary data file 4. All detailed p-values are presented in the supplementary data file 5.

Field-specific reporting

Please select the one below that is the best fit for your research. If you are not sure, read the appropriate sections before making your selection.

☒ Life sciences ☐ Behavioural & social sciences ☐ Ecological, evolutionary & environmental sciences

For a reference copy of the document with all sections, see nature.com/documents/nr-reporting-summary-flat.pdf

Life sciences study design

All studies must disclose on these points even when the disclosure is negative.

Sample size

For the human study, the sample size was determined according to previous studies from the lab (Place et al, PNAS. 2015; Schlittler et al, EJAP. 2019).
No sample-size calculations were performed for the experiments on cells and mice. But according to recent publications in Nature Communications, for Ca2+ measurements, signalling pathways and western blot gels analyses, we performed at least n = 3 independent biological experiments repeats for the cells and collected data from 3 mice for each condition (n =3) to allow statistical analysis and robust conclusions to be drawn.

Data exclusions

All data analysed were included in the statistical analysis. Data were exclused only when clear technical issues arose. When that was the case, the whole experiment was excluded and the experiments were repeated. For example, as the OXPHOS antibody used in the paper is a cocktail antibody, if all the protein complexes did not appear on the gel for any reason, this gel was not considered for quantification. We manage to run each experiment with all the shown conditions in each panel to allow robust comparisons.

Replication

Replicate experiments were successful.

Human data: neuromuscular data were collected as routinely performed in our laboratory. Maximal voluntary contraction (whose reduction after exercise is considered the gold standard to quantify muscle fatique) were repeated two or three times at the start of each experiment to ensure high reproducibility (< 5% different between two consecutive trials). We have shown in previous publications (e.g. Place et al. Muscle Nerve 2007) that the variables used in the present study are reproducible to quantify the origin and the extent of neuromuscular fatigue. For the western blots, muscle biopsies from the each subject of each group (SIT and MICT) at the three time points (Pre, Post and 24h Post) were analyzed together per gel to avoid any technical bias of data collection. Each gel containing a set of SIT and a set of MICT samples was analysed together.

For experiments using differentiated cells: results are composed of at least 3 independent biological experiments, with controls and stimulated or treated groups of cells. Data were collected simultaneously for all conditions assayed/represented on the graphs for each independent biological experiment. All the data were reproducible and are expressed as mean +/- standard deviations, with individual values shown as dots on the bar graphs. Rarely, when technical issues occured, all data from those experiment sets were discarded. The whole experiment was excluded and repeated.

Randomization

Each participant for the human study was randomly assigned to a SIT vs. MICT group. A pairwise randomization was used (i.e. two individuals with comparable VO2max values were randomly allocated to the SIT or the MICT group) to obtain two groups with comparable mean VO2max.
For experiments using differentiated cells, the different plates were randomly assigned to different stimulation protocols.

Blinding

Human experiments: blinding was not possible given the different nature of both exercises being performed.
Cell culture: Blinding was not possible as different samples per group were analysed at the same time for the different technical protocols and it was important to represent each experimental condition when performing sample processing and analyses, and include as much as possible equal number of samples per group for all experimental steps. Moreover, the blinding was not relevant because the operators cannot predict the results before several steps of data analyses.

Reporting for specific materials, systems and methods

We require information from authors about some types of materials, experimental systems and methods used in many studies. Here, indicate whether each material, system or method listed is relevant to your study. If you are not sure if a list item applies to your research, read the appropriate section before selecting a response.

Materials & experimental systems

n/a	Involved in the study
☐	☒ Antibodies
☐	☒ Eukaryotic cell lines
☒	☐ Palaeontology and archaeology
☐	☒ Animals and other organisms
☐	☒ Human research participants
☒	☐ Clinical data
☒	☐ Dual use research of concern

Methods

n/a	Involved in the study
☒	☐ ChIP-seq
☒	☐ Flow cytometry
☒	☐ MRI-based neuroimaging

Antibodies

Antibodies used

Antibodies, Supplier name, Catalogue number, Clone name and number ,Lot number, Dilution used, Validation process, website link

Rabbit anti RyR1, Prof. Marks lab USA, 5029, Polyclonal Aa 1327-1339 10401, 1/5000, RyR1 transfected vs non transfected HEK293 cells

Rabbit phospho RyR1 Ser 2844, Prof. Marks lab USA, "Polyclonal
CRTRRI-(pS)-QTSQ" 10125, 1/5000, Serine to Alanine 2844 knock-in mouse tissues

Rabbit anti S Nitrosocysteine, abm, Y061263, "Polyclonal
Nitrosylated-Cysteine-KLH (hemocyanin)" GR3281561-3, 1/2000, Nitrosylated Cysteine-BSA as a positive control, https://www.abmgood.com/anti-s-nitrosocysteine-sno-cys-produced-in-rabbit-antibody-y061263.html

Rabbit anti DNP, Millipore ,S7150, "Polyclonal
Carbonyl group" 3303032, 1/2000 ,1mM DTT treated cells, https://www.merckmillipore.com/CH/de/product/OxyBlot-Protein-Oxidation-Detection-Kit,MM_NF-S7150?ReferrerURL=https%3A%2F%2Fwww.google.com%2F

Rabbit anti FKBP12, Abcam, ab2918, "Polyclonal
aa 1-13 (N terminal), GVQVETISPGDGR" GR-3312793-1, 1/2500, Elution with Glycine vs IP and Supernatant as negative control, https://www.abcam.com/fkbp12-antibody-ab2918.html

Mouse anti DHPR, Abcam, ab2864, monoclonal 20A IgG2A GR318260-12, 1/1000, ctrl pos: WB: HeLa cell lysate; Rabbit skeletal muscle membrane preparations, https://www.abcam.com/calcium-channel-l-type-dhpr-alpha-2-subunitcacna2d1-antibody-20a-ab2864.html

Rabbit anti SERCA-2a, Abcam, ab150435, monoclonal EPR9392 IgG GR3247974-5, 1/1000, ctrl pos: WB: HeLa and HepG2 whole cell lysates. Rat and mouse brain tissue lysates, https://www.abcam.com/serca2-atpase-antibody-epr9392-ab150435.html

Rabbit anti SERCA-2a, Abcam, ab137020, "Monoclonal
aa 1000-1100 (C terminal)" GR104559-11, 1/1000 ,A673- Hela and HepG2 cells, https://www.abcam.com/serca2-atpase-antibody-epr9393-ab137020.html

MOUSE SERCA1, Abcam, ab2819, monoclonal VE121G9 IGg1 GR3556401-1, 1/1000, KO cell lines,
https://www.abcam.com/serca1-atpase-antibody-ve121g9-ab2819.html

Mouse anti OXPHOS cocktail, Abcam, ab110413, CI subunit NDUFB8(ab110242) monoclonal, 20E9DH10C12,IgG1, Kappa Q5039, 1/1000, "KO cell lines ctrl pos: Isolated mitochondria from Human heart, bovine heart, rat heart and mouse heart. Skeletal muscle tissue, " https://www.abcam.com/ndufb8-antibody-20e9dh10c12-ab110242.html
CII-30kDa (ab14714) monoclonal, 21A11AE7,IgG2A, Kappa KO cell lines ctrl pos: WB: Human, bovine, rat, and mouse heart mitochondria; Isolated mitochondria from HepG2 cells. https://www.abcam.com/sdhb-antibody-21a11ae7-ab14714.html
CIII-Core protein 2 (ab14745) monoclonal, 13G12AF12BB11, IgG1, Kappa KO cell lines ctrl pos: Human, bovine, murine and rat heart mitochondria. HepG2 cell lysate. https://www.abcam.com/uqcrc2-antibody-13g12af12bb11-ab14745.html
CIV subunit I (ab14705) monoclonal, 1D6E1A8, IgG2A, Kappa KO cell lines ctrl pos: WB: Mouse, human, bovine and rat heart mitochondria lysate. https://www.abcam.com/mtco1-antibody-1d6e1a8-ab14705.html
CV alpha subunit (ab14748) monoclonal, 15H4C4, IgG2B, Kappa "KO cell lines ctrl pos: WB: Isolated mitochondria from human, cow, rat and mouse heart. Human liver tissue lysate.
HepG2 whole cell lysate " https://www.abcam.com/atp5a-antibody-15h4c4-mitochondrial-marker-ab14748.html

Mouse anti PGC-1, Sigma, ST1202, monoclonal, 4C1.3, IgG 3520161, 1/1000, Whole cell lysates from COS cells transformed with wild-type PGC-1α (5 µg, lane 1; 10 µg, lane 2; 20 µg whole cell extract from brown adipose tissue of a cold-exposed mouse, and whole cell

lysate from CHO cells transformed with NT-PGC-1α (0.5 μg), https://www.merckmillipore.com/CH/fr/product/Anti-PGC-1-Mouse-mAb-4C1.3,EMD_BIO-ST1202?bd=1#anchor_COA

Rabbit anti NRF1, Abcam, ab175932, monoclonal EPR5554(N),IgG GR247239-10 ,1/1000, "ctrl pos: WB: MCF-7, HeLa and 293T cell lysates and human, fetal heart, mouse heart, mouse brain, rat heart and rat brain tissue lysates " https://www.abcam.com/nrf1-antibody-epr5554n-chip-grade-ab175932.html

Rabbit anti Tfam (mouse muscle) Abcam ab131607 "polyclonal IgG, Recombinant full length protein (His-tag) corresponding to Mouse mtTFA. Native recombinant mouse mtTFA protein with a C-terminal 6-His tag" GR259208-9 1/1000 KO cell lines ctrl pos:Rat kidney lysate https://www.abcam.com/mttfa-antibody-mitochondrial-marker-ab131607.html

Mouse anti Tfam (human muscle), Abcam, ab119684, monoclonal 18G102B2E11, IgG2B, Kappa GR68222-14 ,1/1000, KO cell lines, https://www.abcam.com/mttfa-antibody-18g102b2e11-mitochondrial-marker-ab119684.html

Mouse anti OPA1, BD-Biosciences, 612607 IgG1, 18/OPA1 7201874, 1/1000, "Western blot analysis of OPA1 on a K-562 cell lysate (Human bone marrow myelogenous leukemia; ATCC CCL-243), " https://www.bdbiosciences.com/en-eu/products/reagents/microscopy-imaging-reagents/immunofluorescence-reagents/purified-mouse-anti-opa1.612607

Rabbit anti DRP1, Abcam, ab184247, monoclonal EPR19274, IgG GR3254224-3, 1/1000, "WB: Human fetal kidney, rat brain, rat heart and mouse brain lysates; A549, U-2 OS, HeLa, Jurkat, HEK-293, HCT 116, PC-12 and NIH/3T3 whole cell lysates" , https://www.abcam.com/drp1-antibody-epr19274-ab184247.html

Rabbit anti-Pyruvate dehydrogenase phospho serine 293, Abcam, ab177461, monoclonal EPR12200, IgG GR3281506-4, 1/1000, "ctrl pos: WB: Human fetal kidney, rat brain, rat heart and mouse brain lysates; A549, U-2 OS, HeLa, Jurkat, HEK-293, HCT 116, PC-12 and NIH/3T3 whole cell lysates", https://www.abcam.com/pdha1-phospho-s293-antibody-epr12200-ab177461.html

Mouse anti total Pyruvate dehydrogenase, Abcam, ab110334, monoclonal 8D10E6, IgG1 Kappa GR3332330-1, 1/1000, "WB: Isolated mitochondria from human, bovine, rat and mouse heart. HepG2 (human liver hepatocellular carcinoma cell line) cell lysate. KO cell lines", https://www.abcam.com/pdha1-antibody-8d10e6-ab110334.html

Rabbit anti phospho AMPKa threonine 172, Cell Signaling, 2535S, monoclonal IgG, 40H9 21, 1/1000, Western blot analysis of extracts from C2C12 cells, untreated or oligomycin-treated (0.5 μM), https://www.cellsignal.com/products/primary-antibodies/phospho-ampka-thr172-40h9-rabbit-mab/2535?site-search-type=Products&N=4294956287&Ntt=2535s&fromPage=plp&_requestid=2536764

Mouse anti total AMPKa, Cell Signaling, 2793, IgG2B, F6 7, 1/1000, Western blot analysis of extracts from various cell types using AMPKα (F6) Mouse mAb, https://www.cellsignal.com/products/primary-antibodies/ampka-f6-mouse-mab/2793

Rabbit CaMKII phospho threonine 286, Cell Signaling, 12716S, monoclonal IgG, D21E4 5, 1/1000, Western blot analysis of extracts from MKN-45 cells treated with λ-phosphatase or Forskolin #3828 (30 μM, 20 min). Western blot analysis of extracts from rat brain, mouse brain and human cerebellum. Western blot analysis of extracts from 293T cells, untransfected or transfected with specific CamKII proteins . Antibody phosphospecificity was verified by preincubating the antibody in the absence of a peptide (-) or with either CamKII-β (Thr287) phosphopeptide (+) or CamKII-β (Thr287) non-phosphopeptide (+) prior to incubating the membrane, https://www.cellsignal.com/products/primary-antibodies/phospho-camkii-thr286-d21e4-rabbit-mab/12716?site-search-type=Products&N=4294956287&Ntt=12716s&fromPage=plp&_requestid=2544294

Rabbit CaMKII pan, Cell Signaling, 3362S, Polyclonal antibodies are produced by immunizing animals with a synthetic peptide corresponding to the amino-terminal region of human CaMKII. Antibodies are purified by protein A and peptide affinity chromatography 6, 1/1000, Western blot analysis of extracts from rat and mouse brain using CaMKII (pan) Antibody, https://www.cellsignal.com/products/primary-antibodies/camkii-pan-antibody/3362?site-search-type=Products&N=4294956287&Ntt=3362s&fromPage=plp&_requestid=2544392

Rabbit anti MCU, Sigma, HPA016480, Coiled-coil domain-containing protein 109A recombinant protein epitope signature tag (PrEST) 0,00013816, 1/1000, WB analysis in A-549 cells transfected with control siRNA, target specific siRNA probe nos.1 and 2, using anti-MCU antibody ctrl: anti PPIB RNAi knockdown, https://www.sigmaaldrich.com/CH/fr/product/sigma/hpa016480?context=product

Mouse anti Gapdh, Sigma, G9545, polyclonal Synthetic peptide corresponding to amino acids of mouse GAPDH, conjugated to KLH via an N-terminal cysteine residue. The corresponding sequence is identical in rat and differs by two amino acids in humans. 128M4817, 1/1000 , https://www.sigmaaldrich.com/CH/fr/product/sigma/g9545?context=product

Rabbit sarcolipin, Sigma, ABT13 "polyclonal IgG, KLH-conjugated linear peptide corresponding to the C-terminus of human Sarcolipin." 3143173 1/500 Evaluated by Western Blotting in rabbit quadraceps tissue lysate, https://www.merckmillipore.com/CH/fr/product/Anti-Sarcolipin-Antibody,MM_NF-ABT13?bd=1#anchor_COA

Rabbit STIM1, Sigma, S6072, polyclonal IgG, synthetic peptide corresponding to amino acids 61-74 of human STIM1, conjugated to KLH via a C-terminal cysteine residue. The corresponding sequence is identical in rat and mouse. 104M4822V, 1/1000, NIH-3T3 mouse fibroblasts, https://www.sigmaaldrich.com/CH/de/product/sigma/s6072

IRDye 680- conjugated donkey anti-mouse Li-Cor 926-68022 C90821-12 1/10'000
IRDye 680- conjugated donkey anti-rabbit Li-Cor 926-68023 C70308-05 1/5'000
IRDye 800-conjugated donkey anti-mouse Li-Cor 926-32212 C70502-02 1/10'000

	IRDye 800-conjugated donkey anti-rabbit Li-Cor 926-32213 C90806-09 1/5'000
Validation	All the antibodies used in this manuscript have been previously validated by the suppliers and/or referenced in relevant publications.

Eukaryotic cell lines

Policy information about cell lines

Cell line source(s)	The cell line used in this study is the C2C12 mouse cell line from ATCC provided by Professor Lluis Fajas lab at the University of Lausanne.
Authentication	The C2C12 cells used in this study were authenticated by ATCC and validated through the myotube formation testing.<br><br>Myoblasts from the C2C12 cell line proliferate well, migrate and differentiate into myotubes according to the description from the original ATCC supplier. Cells were always used under passage 13 to avoid lost of differentiation potential.
Mycoplasma contamination	The C2C12 cell line was tested negative for mycoplasma.
Commonly misidentified lines<br>(See ICLAC register)	No commonly misidentified cell lines was used in this study.

Animals and other organisms

Policy information about studies involving animals; ARRIVE guidelines recommended for reporting animal research

Laboratory animals	C57BL/6J (B6) mice obtained from Janvier were provided by the animal facility from the Department of Biomedical Sciences, University of Lausanne. The animals were maintained in a temperature-controlled animal facility with a 12-hour light/12-hour dark cycle and had access to food and water according to the Swiss Animal Protection Ordinance (OPAn). 8-week female mice were used in this study. Our protocol was approved by the animal Ethics Committee of Lausanne (commission cantonale pour l'expérimentation animale) with the number VD3489.
Wild animals	The study did not involve any wild animals.
Field-collected samples	The study did not involve field-collected samples.
Ethics oversight	The study was approved by the animal Ethics Committee of Lausanne (commission cantonale pour l'expérimentation animale) with the number VD3489.

Note that full information on the approval of the study protocol must also be provided in the manuscript.

Human research participants

Policy information about studies involving human research participants

Population characteristics	Participants included in the study were 21-30 years old healthy men, young, recreationally active adults.<br>Our study complies in full with the STROBE statement.
Recruitment	Volunteers were recruited through a recruitment advertisement and were mostly students in sport sciences. Informed consents were obtained from all subjects.
Ethics oversight	The study was validated by the Commission d'éthique de la recherche sur l'être humain du Canton de Vaud (protocol 2017-00303)

Note that full information on the approval of the study protocol must also be provided in the manuscript.
